# Clinical Use of the Self-Assembling Peptide RADA16: A Review of Current and Future Trends in Biomedicine

**DOI:** 10.3389/fbioe.2021.679525

**Published:** 2021-06-02

**Authors:** Sharanya Sankar, Kate O’Neill, Maurice Bagot D’Arc, Florian Rebeca, Marie Buffier, Elton Aleksi, Melanie Fan, Noriaki Matsuda, Eun Seok Gil, Lisa Spirio

**Affiliations:** ^1^3-D Matrix Europe SAS, Caluire-et-Cuire, France; ^2^3-D Matrix UK Ltd., London, United Kingdom; ^3^BLUEPHARM SAS, Paris, France; ^4^3-D Matrix Inc., Newton, MA, United States; ^5^3-D Matrix, Ltd., Tokyo, Japan

**Keywords:** self-assembling peptide hydrogel, nanofiber, RADA16, hemostasis, tissue regeneration, wound healing, surgery, endoscopy

## Abstract

RADA16 is a synthetic peptide that exists as a viscous solution in an acidic formulation. In an acidic aqueous environment, the peptides spontaneously self-assemble into β-sheet nanofibers. Upon exposure and buffering of RADA16 solution to the physiological pH of biological fluids such as blood, interstitial fluid and lymph, the nanofibers begin physically crosslinking within seconds into a stable interwoven transparent hydrogel 3-D matrix. The RADA16 nanofiber hydrogel structure closely resembles the 3-dimensional architecture of native extracellular matrices. These properties make RADA16 formulations ideal topical hemostatic agents for controlling bleeding during surgery and to prevent post-operative rebleeding. A commercial RADA16 formulation is currently used for hemostasis in cardiovascular, gastrointestinal, and otorhinolaryngological surgical procedures, and studies are underway to investigate its use in wound healing and adhesion reduction. Straightforward application of viscous RADA16 into areas that are not easily accessible circumvents technical challenges in difficult-to-reach bleeding sites. The transparent hydrogel allows clear visualization of the surgical field and facilitates suture line assessment and revision. The shear-thinning and thixotropic properties of RADA16 allow its easy application through a narrow nozzle such as an endoscopic catheter. RADA16 hydrogels can fill tissue voids and do not swell so can be safely used in close proximity to pressure-sensitive tissues and in enclosed non-expandable regions. By definition, the synthetic peptide avoids potential microbiological contamination and immune responses that may occur with animal-, plant-, or mineral-derived topical hemostats. *In vitro* experiments, animal studies, and recent clinical experiences suggest that RADA16 nanofibrous hydrogels can act as surrogate extracellular matrices that support cellular behavior and interactions essential for wound healing and for tissue regenerative applications. In the future, the unique nature of RADA16 may also allow us to use it as a depot for precisely regulated drug and biopharmaceutical delivery.

## Introduction to Self-Assembling Peptides (SAPs)

Self-assembling peptides (SAPs) are short oligopeptides that often contain repeating amino acid sequences, which, under appropriate environmental conditions spontaneously self-assemble into distinct nanostructures (Edwards-Gayle and Hamley, 2017; Lee et al., 2019). Explorations into the characteristics and biomedical utility of SAPs followed the discovery in 1989 of a native yeast protein containing repeated amino acid sequences, zuotin, that self-assembled into 3-dimensional (3D) structures in specific fluid environments (Zhang et al., 1992). This process of molecular self-assembly found in nature inspired scientists to develop unique SAPs for research and medical product development, including RADA16 (Zhang, 2017). The SAP monomeric constitution can be purposefully selected so that spontaneous self-assembly forms distinct well-ordered supramolecular structures with desired configurations including sheets, vesicles, tubes, and interlinking fibrous networks resembling natural extracellular matrix (ECM). Self-assembly of SAP peptide molecules into discrete structures primarily involves non-covalent interactions including hydrogen bonding, electrostatic interactions, hydrophobic/hydrophilic relationships, and van der Waals forces (Edwards-Gayle and Hamley, 2017).

Various subclasses of SAPs have been identified and characterized (Hartgerink et al., 2001; Chen, 2005; Choi et al., 2012; Argudo et al., 2018; Qiu et al., 2018). One well-studied SAP subgroup are the ionic-complementary SAPs, characterized by an alternating sequence of positively and negatively charged amino acids (Chen, 2005). Ionic-complementary SAPs are further categorized by specific residue charge distribution patterns into Type I, +−+−; Type II, ++−−; Type III, +++−−−; or Type IV, ++++−−−− monomers, with the subclass defined by the number of similarly charged peptides occurring in sequence. Type-1 ionic-complementary peptides have β-strand periodicity and their regular charge patterns allow their spontaneous interlinking assembly by hydrophobic effect and hydrogen bonding into predictable complex β-sheet suprastructures. The specific charge pattern of SAP peptides determines their interlocking assembly configuration, allowing scientists to generate different polymeric structures with distinct biological behavior and interactions. This review focuses on one such Type-I peptide, RADA16-I (hereafter referred to as RADA16), a SAP that spontaneously self-assembles into nanofibrous structure and that under physiological conditions forms a complex hydrogel of interwoven networks. RADA16 has demonstrated clinical utility as a highly effective hemostatic agent for controlling surgical bleeding and shows great promise as a surrogate ECM for wound healing and tissue regeneration, and as an efficient drug delivery platform.

## Mechanism of Action of RADA16

**FIGURE 1:**
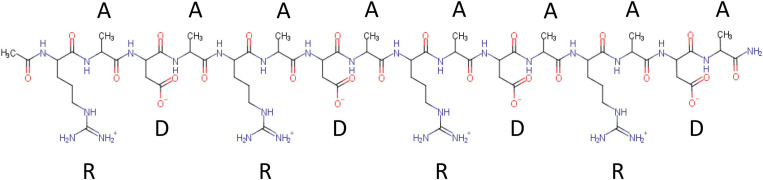
Chemical structure of RADA16 peptide. RADA16 peptide chemical structure showing the 16 amino acids organized as sequentially repeated 4-amino acid sequences containing R (positively charged arginine), A (hydrophobic alanine), and D (negatively charged aspartic acid) residues.

RADA16 is a 16-amino acid Type I-SAP containing repeated R (positively charged arginine), A (hydrophobic alanine), and D (negatively charged aspartic acid) amino acid residues (Figure 1). A single RADA16 peptide is a ∼6 nm-length monomeric oligopeptide.

**FIGURE 2:**
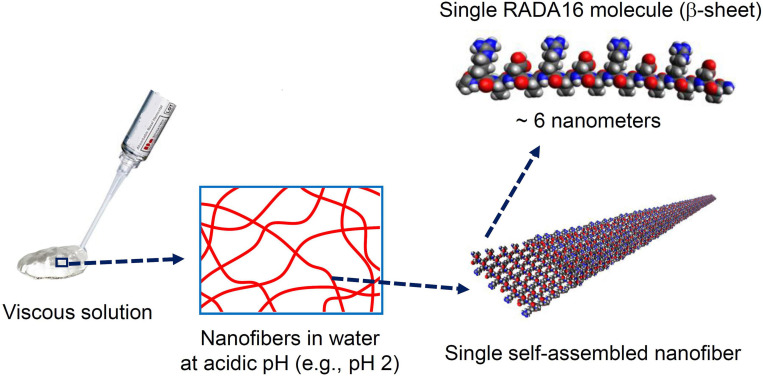
Macro, micro, and nanostructures of RADA16 in an aqueous solution. Spontaneous and reversible self-assembly of RADA16 molecules occurs in acidic solutions to generate nanofibers. RADA16 molecules with β-sheet conformation interact through face-to-face hydrophobic interactions and edge-to-edge hydrogen bonding to form layered and extended nanofibers, ∼6 nm in width. These ECM-like nanofibers form a viscous and transparent aqueous solution at a relatively low concentration range (e.g., 0.1∼2.5% weight/volume).

**FIGURE 3:**
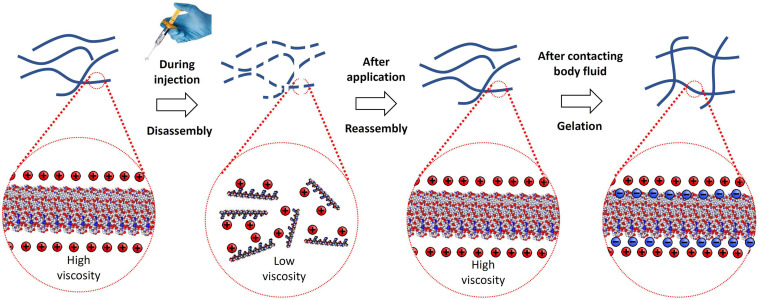
Illustration of RADA16 structure and properties as it is applied to and gels on a wound site. Acidic aqueous solutions of RADA16 are viscous and exhibit shear-thinning and thixotropic disassembly/reassembly, which allows their easy administration to wound sites through catheters and syringes with viscosity returning immediately after administration. Upon contact with the physiological pH of body fluids including blood and interstitial fluid, the surface net charges of RADA16 nanofibers become zero resulting in the physical crosslinking by hydrophobic interactions between neighboring RADA16 nanofibers, so that RADA16 solution forms *in situ* hydrogels on the wound site and act as a physical barrier to bleeding.

RADA16 peptides are manufactured in an acidic environment (pH ≈2) and spontaneously self-assemble to form nanofibers in water. At pH 2, the net charge of a RADA16 peptide is positive because the side carboxylic acids of its four aspartic acids are protonated in water (i.e., no charge under their pKa of ≈3.7) while the side guanidine groups of its four arginines are positively charged. The presence of hydrophobic amino acid groups and positively charged sequences along the RADA16 peptide allows monomer organization into stable β-sheet nanofiber with a positively charged surface in this aqueous acidic environment (Figure 2; Yokoi et al., 2005). Therefore, the RADA16 nanofibers are flowable in an acidic aqueous condition due to electric repulsion among the positively charged nanofibers, while they generate high viscosity due to their fibrous structure. The diameter of the RADA16 nanofibers is smaller than the wavelength of visual light (i.e., 380–740 nm) so the solution appears transparent. The action of self-assembly is spontaneous and reversible, so the peptides can disassemble upon exposure to external shearing force and spontaneously reassemble after removal of the force (Figure 3). This property allows for easy application and coverage of wounds due to partial disassembly of RADA16 peptides as the material is applied but its viscosity immediately returns after application via molecular reassembly. In clinical practice, these shear-thinning and thixotropic properties make RADA16 solutions suitable for application through a narrow applicator and allow the product to flow easily into wounds (Figure 3).

When the peptide solution is neutralized and buffered by the physiological pH and ionic characteristics of bodily fluids (e.g., blood, lymph, cerebrospinal fluid) or tissue culture media, the side carboxylic acids of the RADA16 four aspartic acids are deprotonated (i.e., negatively charged over their pKa of ≈3.7, which creates both four positive charges and four negative charges on the RADA16 peptide, and the net charge becomes zero). The surface of the nanofibers becomes hydrophobic; thus, the neighboring nanofibers are physically cross-linked via hydrophobic interactions to become a complex mesh-like hydrogel structure (Figure 3) that resembles native ECM architecture (Yokoi et al., 2005; Cormier et al., 2013; Zhang, 2017; Wang et al., 2019).

These unique properties of RADA16 have been adapted by researchers and surgeons for diverse experimental and clinical applications. RADA16 has been commercialized as PuraMatrix^TM^ (3-D Matrix Ltd., Tokyo, Japan), a 1% peptide solution for use as an ECM surrogate for laboratory evaluation of cell adhesion, chemotaxis, proliferation and development, and as an *in vitro* and pre-clinical *in vivo* 3D scaffold for hemostasis, wound healing, and tissue engineering investigations (Wang et al., 2019; Corning Life Sciences Inc, 2021).

**FIGURE 4:**
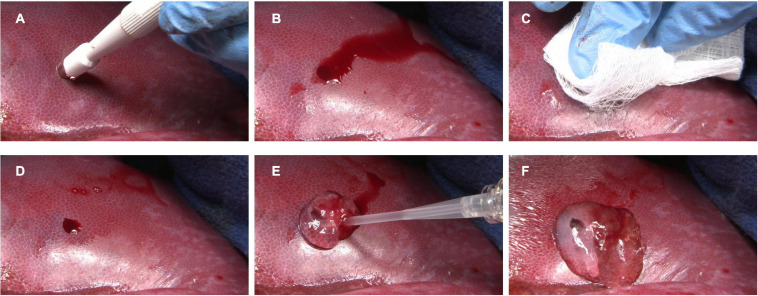
Hemostatic use of RADA16 self-assembling peptide hydrogel to control bleeding from wound surfaces of the liver. In a porcine model, punch biopsy of the liver surface **(A)** results in frank bleeding **(B)**. After site irrigation and drying **(C)**, residual bleeding **(D)** is stopped by the easy single-syringe application of RADA16 solution **(E)**, which rapidly forms a transparent hemostatic *in situ* hydrogel barrier upon contact with physiological fluids **(F)**. This approach can be used to stop topical bleeding at surgical and wound sites in skin, organs, vessels, and other tissues.

**FIGURE 5:**
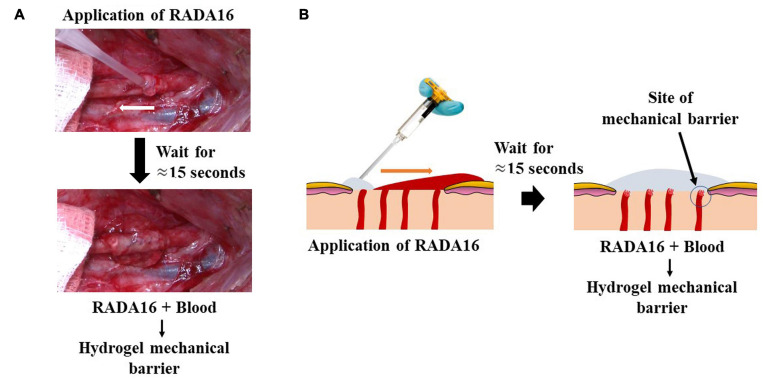
Hemostatic mechanism of action of RADA16 self-assembling peptide hydrogel on bleeding wound surfaces. Upon contact with physiological fluids present at the surgical site, the RADA16 solution rapidly forms a transparent hemostatic *in situ* hydrogel barrier on the oozing suture lines of vascular anastomoses **(A)** and the topical/surgical oozing sites such as ulcer and resected tissue after surgery **(B)**. In a porcine model using the femoral artery, a longitudinal suture line was conventionally irrigated and sponge-dried, and RADA16 was syringe-applied to the suture line on the vessel’s outer surface. The white arrow shows the direction of syringe movement during application. RADA16 solution can stop residual bleeding at blood vessel suture lines and anastomoses sites during cardiothoracic and vascular surgeries **(A)**. In a general topical/surgical bleeding site, *in situ* hemostatic hydrogel formation is initiated at the interface between the bleeding site and the applied RADA16 layer. This is represented diagrammatically **(B)**. The transparent nature of the hydrogel allows easy visualization of the surgical field and underlying sutures and bleeding sites, thus enabling the surgeon to evaluate the surgical site for satisfactory hemostasis and the possible need to perform revisions.

In the clinic or operating room, the SAP products PuraStat^®^ and PuraBond^®^ (3-D Matrix Europe SAS, Caluire-et-Cuire, France), are delivered by pre-filled syringes as viscous aqueous solutions of synthetic 2.5% RADA16, that spreads across the surface of the wound. When RADA16 comes in to contact with blood or other physiological fluids, it forms a hydrogel. The hydrogel formation acts as a barrier and blocks the flow of the blood from the wound and thereby, demonstrates excellent topical hemostatic control of intra- and post-operative bleeding associated with diverse surgical procedures, including oozing from wound surfaces of the skin and other organs (Figure 4) and from suture lines of vascular anastomoses (Figure 5; 3-D Matrix Europe SAS, 2014; Wang et al., 2019). The hemostatic mechanism of action of the RADA16 products is *in situ* formation of a transparent hemostatic hydrogel barrier on bleeding wound surfaces upon contact with physiological fluids present at the surgical site (Figure 5). A sister product cleared in the United States, PuraSinus^TM^ (also 2.5% RADA16), is an intraoperatively applied hemostatic wound dressing that also prevents adhesion formation and acts as an adjunct to wound healing after nasal surgery or trauma (US Food and Drug Administration, 2021). Finally, PuraDerm^TM^ has been cleared in the United States as topical wound dressing for the management of partial and full-thickness wounds such as pressure sores, leg ulcers, diabetic ulcers, and surgical wounds.

One other SAP is currently approved for clinical use by regulatory authorities besides RADA16, an 11 amino acid peptide (QQRFEWEFEQQ) known as PF_11_-4 (commercial name Curodont^TM^ Repair) that promotes remineralization and regression in early dental caries (Alkilzy et al., 2018; Sedlakova Kondelova et al., 2020). This review focuses on the evolution of applying RADA16 for surgical bleeding control and provides an overview of studies indicating the great potential of this unique formulation in facilitating wound healing, tissue regeneration, and as a drug-delivery depot.

## Topical Hemostatic Agents for Surgical Bleeding

Intraoperative and post-operative bleeding is a risk of all surgical procedures (Dagi, 2005), and appropriate treatment depends upon the site, cause, and extent of blood loss. Ongoing rapidly evolving technological advances have provided surgeons with an expanding diversity of innovative approaches for achieving intraoperative and post-operative hemostasis in their patients. Standard hemostatic methods range from simple pressure application, electrocautery, vessel ligation, and suturing for primary wound closure, intravenous administration of blood products, and systemic or topical delivery of procoagulation agents.

Modern biotechnology has developed many new topical hemostats for surgical use, including formulations based on oxidized cellulose, gelatin, collagen, fibrin and thrombin, hyaluronic acid, and cyanoacrylates. All of these topical hemostatic classes have unique strengths and limitations (Achneck et al., 2010; Pereira et al., 2018). An ideal topical hemostatic agent would be easy to administer in diverse surgical and post-operative scenarios, rapid-acting, effective, completely biocompatible, non-animal derived, resorbable, easy to use, and cost-effective. A newer class of self-assembling topical hemostats including RADA16 may best satisfy all of these requirements.

## Surgical Hemostasis Using RADA16

**FIGURE 6:**
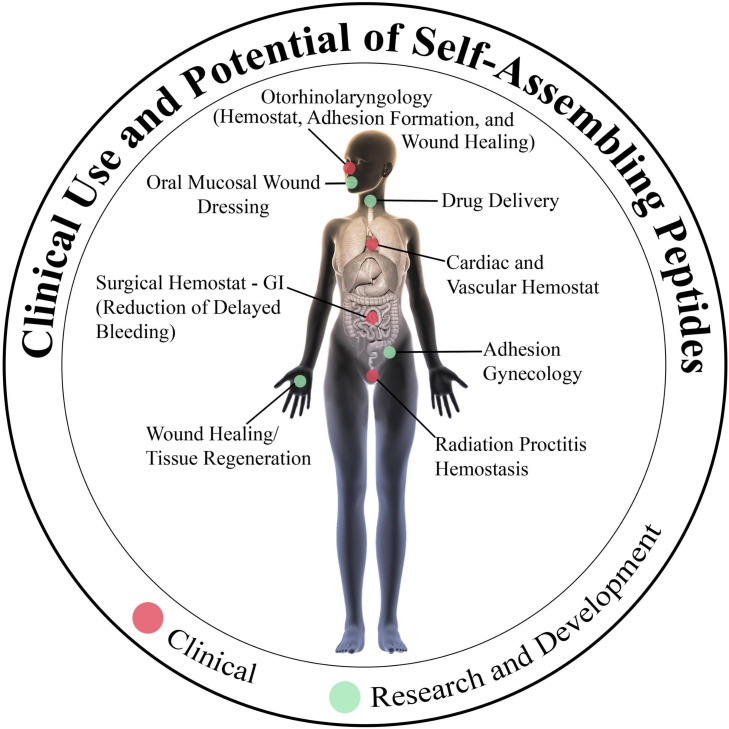
Current and future clinical uses of the self-assembling peptide RADA16. Hemostatic RADA16 formulations are currently in use for stopping intraoperative bleeding and preventing delayed/rebleeding in cardiovascular, gastrointestinal, and otorhinolaryngological surgical procedures. Experimentation is underway to determine if RADA16 can act as a surrogate extracellular matrix to improve wound healing and tissue regeneration in diverse tissues and organs, and possibly act as a precisely regulated drug delivery depot.

PuraStat and PuraBond are 2.5% RADA16 formulations that are CE-marked as Class III medical devices for hemostatic use in humans. They are indicated as adjunctive hemostatic supplements to intraoperative ligation and suturing, to control exudative bleeding from small blood vessels and parenchyma of solid organs, at vascular anastomoses, and in the case of PuraStat from small vessels of the gastrointestinal (GI) tract mucosa following endoscopic and laparoscopic tissue resection (Figures 4–6; 3-D Matrix Europe SAS, 2014). A related product, PuraSinus (also a 2.5% aqueous RADA16 formulation), was cleared by the FDA in 2019 as an intraoperatively applied wound dressing for achieving hemostasis, preventing adhesion formation, and as an adjunct to wound healing after nasal surgery or trauma repair (Figure 4–6; US Food and Drug Administration, 2021).

Sterile-filtered acidic RADA16 hemostatic solutions exhibit long-term stability during storage at refrigerator temperatures (3-D Matrix Europe SAS, 2014; US Food and Drug Administration, 2021). Syringe delivery of SAP solutions facilitates easy one-handed application in most circumstances, without the need to mix or dissolve components before application as with some fibrin-based hemostats (Spotnitz, 2010), for example. Thus, it is “ready to use,” with no preparation requirements or complicated handling that may prove cumbersome for surgeon or nurses.

The RADA16 aqueous formulations can be precisely applied within tight surgical fields; their fluidity conforms to irregular tissue surfaces, and their viscosity allows filling tissue voids and prevents unwanted migration away from the application site. Unlike some topical hemostats, RADA16 does not absorb bodily fluids and expand in volume after application, which avoids the risk of compressive injury when used in pressure-sensitive tissues or physically constrained structures such as the coronary arteries and nerves (Schonauer et al., 2004; Tomizawa, 2005; Pereira et al., 2018). The RADA16 hydrogel remains transparent even after gelation (Figures 4, 5), so clear visualization of the surgical site and suture lines is maintained. This facilitates continuous surveillance of the operative area to evaluate the need for wound closure repair or revision, because any residual bleeding is instantly identifiable. Hemostats based on RADA16 can be used in combination with electrosurgical coagulation forceps, and clips and sutures may be placed through the hydrogel after application. Prophylactic administration of RADA16 solutions can also be used to reduce the likelihood of delayed-onset bleeding after surgical procedures such as endoscopic resection (Subramaniam et al., 2019).

Gelation of applied aqueous RADA16 begins upon contact with physiological fluid which buffers the acidic aqueous solution and provides an appropriate ionic microenvironment for hydrogel formation (Ellis-Behnke et al., 2006a; Wang et al., 2012). Exposure to tissue interstitial fluids in the absence of frank bleeding will also initiate RADA16 gelation (Subramaniam et al., 2019). Compared to animal- and plant-derived hemostats such as collagen and cellulose agents, pure synthetic SAP formulations such as RADA16 carry no risk of containing unwanted contaminants that may be pyrogenic or present other safety concerns (Genove et al., 2005). The RADA16 3D meshwork pore sizes are small enough (50–200 nm) to restrict platelet (2–3-μm diameter) and erythrocyte (6–8-μm diameter) penetration and achieve hemostasis. Instead, the blood-contacting RADA16 solution begins rapid gelation (as quickly as 15–20 s) from its surface and presents a hemocompatible surface for platelets and erythrocytes to accumulate on and initiate hemostasis, thereby enhancing the hemostasis achieved by the RADA16 matrix’s physical barrier function alone (Figure 5; Saini et al., 2016; Taghavi et al., 2018). The hydrogel components themselves, however, do not appear to directly activate either platelets or complement C3a/C4, but do provide a substratum upon which physiological coagulation reactions, likely initiated by wound-released factors, may proceed (Saini et al., 2016; Taghavi et al., 2018). The PuraStat matrix contains 97.5% water, which allows the unhindered diffusion of soluble nutrients, growth factors, and oxygen that are essential mediators of healing at the wound surfaces.

Unlike some topical hemostatic agents, neither RADA16 hydrogel nor its constituents induce inflammatory or immune reactions (Genove et al., 2005; Wang et al., 2019). Over time, applied PuraStat and PuraSinus^TM^ are degraded to their constituent natural and entirely biocompatible L-amino acids by endogenous proteolytic/hydrolytic mechanisms, the fragments of which are then either metabolized or recycled, ultimately removing all residual RADA16 from the surgical site.

Topical hemostatic formulations should be convenient to administer in diverse operative settings, act quickly to efficiently staunch bleeding, be non-allergenic and non-inflammatory, facilitate and not inhibit wound healing, be resorbable, and cost-effective. Members of the SAP family such as RADA16 may best fulfill these criteria, and eventually become a standard-of-care for controlling and preventing mild-to-moderate bleeding associated with many surgical scenarios.

Indeed, following the approval of RADA16 for clinical use as a topical hemostatic agent, its utility has been demonstrated in multiple surgeries that are commonly performed in, for example but not limited to, cardiothoracic and vascular surgery, gastroenterology, and otorhinolaryngology specialties (Figure 6).

### RADA16 Hemostasis in Cardiac and Vascular Surgery

After demonstrating excellent hemostasis with 2–2.5% RADA16 solutions (from 3-D Matrix Inc.) in pre-clinical models of rabbit aortic puncture and dog aortic graft anastomoses, RADA16 was evaluated for safety and hemostatic effectiveness in human subjects undergoing cardiovascular surgery (Masuhara et al., 2012). Subjects comprised 25 individuals (22 male/3 female; age 54–80 years), who underwent coronary artery bypass grafting (*n* = 9), abdominal aortic graft replacement (*n* = 4), or peripheral artery bypass (*n* = 12) at two Japanese medical centers. The target sites for 2.5% RADA16 application were mild-to-moderate remnant bleeding at vessel-to-vessel anastomoses, graft anastomoses, and autologous vein patch plasty sites; other hemostatic approaches were employed if bleeding was copious or spurting. All subjects had been heparinized during surgery, which was reversed by protamine sulfate injection immediately after RADA16 application. Of 33 total bleeding sites, the mean application area was 3.3 cm^2^ using an average of 1.5 mL (range 0.5–3.0 mL) RADA16 solution at each site. Approximately 1 mL of RADA16 was applied to coronary anastomoses, 2 mL to aortic anastomoses, and 1 mL to other peripheral vascular anastomotic sites. The immediate hemostasis effectiveness rate was 88% (29/33 sites), and mean hemostasis time was 154 ± 39 s. Effectiveness was 100% if a second treatment was used to treat post-operative bleeding. No RADA16-related safety issues were observed. The researchers concluded that RADA16 is an efficacious hemostatic agent for stopping oozing bleeding in cardiovascular surgery.

The safety, performance, and ease of use of RADA16 (PuraStat) as a hemostatic agent during left ventricular assist device (LVAD) implantation was evaluated in a prospective study of 15 patients at a single center in Germany (Morshuis et al., 2019). Application sites included the apical cannulation, and the outflow graft anastomoses. Hemostatic effectiveness was confirmed in 93% (27/29) of evaluated bleeding sites, with a rapid average time to hemostasis of 19.4 ± 13.0 s. There were no RADA16-related adverse events (AEs), and no inflammation, excessive granulation, or foreign body reaction was observed through post-operative 24 months.

Thoracic surgeons were queried about experiences using RADA16 for hemostasis in 50 consecutive cardiovascular surgery patients (mean age 72 years) at two medical centers in the United Kingdom who underwent various procedures including coronary artery bypass, valve repair or replacement, sometimes with grafting, and aortic repair or replacement (Giritharan et al., 2018). The most common application sites for RADA16 were aortotomy closures (62%) and graft suture lines (18%). Other areas of RADA16 application included oozing bleeding at cannulation sites, patch repairs, needle hole bleeds from prosthetic grafts, and at the top end anastomosis of vein grafts during coronary artery bypass. Surgeons rated the syringe-delivered product as easy-to-use, even in hard-to-reach surfaces. Hemostasis was achieved with RADA16 alone in 84% of applications and it worked well in conjunction with other hemostatic agents (e.g., Bioglue^®^ and Fibrillar^®^). The distinctive transparent nature of PuraStat was appreciated because it allowed clear visualization of suture lines after its application. Mean blood product use of packed red cells, platelets, fresh-frozen plasma, and cryoprecipitate was below the national average for these procedures. No RADA16-related AEs were reported.

### RADA16 Hemostasis in Gastrointestinal Endoscopic Surgery

Intraprocedural bleeding (IPB) and delayed bleeding or rebleeding are significant challenges encountered during endoscopic resection of lesions or endoscopic intervention for acute GI bleeding (Kataoka et al., 2016). Current standard treatment modalities such as electrocoagulation, argon plasma coagulation, and mechanical clip placement pose a risk of thermal injury or perforation and can be technically challenging depending on the bleeding site and endoscopist experience (Chetcuti Zammit and Sidhu, 2018). Recently, topical hemostatic powders have been introduced as additional options for managing bleeding in the GI tract (Mourad and Leong, 2018; de Rezende et al., 2019). However, these powders do not precisely cover the target lesion, but typically are dispersed well beyond the margins of the bleeding defect. Additionally, existing hemostatic powders are opaque, which can block the surgeon’s view of the operative field and hinder evaluation of the bleeding source and are prone to premature gel formation that may clog the catheter (de Nucci et al., 2020). The bleeding in the GI tract can also be treated with RADA16-based hemostats, which can staunch unwanted bleeding while the transparent nature of the hydrogel maintains excellent visualization of the surgical field, and premature gelation within the catheter does not occur (Yoshida et al., 2014; Pioche et al., 2016; Subramaniam et al., 2019).

The hemostatic utility of RADA16 was reported in 12 subjects who experienced oozing bleeding during interventional endoscopic mucosal resection (EMR) or endoscopic submucosal dissection (ESD) for gastric tumors (Yoshida et al., 2014). Hemostasis with RADA16 was deemed “remarkably effective” in 11 patients and “effective” in 1 patient. Time-to-hemostasis was 105 ± 87 s, using an average of 3.3 ± 2.1 ml of RADA16 per lesion. No secondary hemorrhages occurred in any subject. Following this initial proof-of-concept study that indicated good feasibility and effectiveness, these surgeons indicated the need to perform further studies involving a larger number of patients in Japan and in Europe.

A study at two centers in France evaluated the hemostatic effectiveness of RADA16 on delayed bleeding in 56 subjects (age 67 ± 11 years) with 65 diverse GI lesions (22 stomach, 15 rectum, 10 duodenum, 8 esophagus, 7 colon, and 3 ampullary) (Pioche et al., 2016). Forty patients underwent ESD procedures and 18 EMR procedures. The rate of delayed bleeding after RADA16 use was only 6% despite 45% of lesions (29/65) having been considered at high-risk of rebleeding. No device-related AEs occurred, and endoscopists reported easy administration of the peptide solution through the catheter and continuous visualization of the resection bed.

The effectiveness of RADA16 was evaluated for preventing delayed bleeding in 45 consecutive patients (51 lesions) who underwent gastric ESD in Japan (1% RADA16 was used, termed PuraMatrix by the authors) (Uraoka et al., 2016). RADA16 was applied to the resected base at the end of the procedure. The rate of post-ESD bleeding was 2% (1/51 lesions), defined as any case requiring endoscopic or surgical intervention or having a decrease in hemoglobin level of 2 g/dL. Endoscopic follow-up was performed at 1, 4, and 8 weeks after ESD and no AEs related to RADA16 occurred. The rate of healing at week 1 and the rate of scarring at weeks 4 and 8 were higher than rates reported in the literature, suggesting that the peptide facilitated ulcer healing.

Safety, effectiveness, and technical feasibility of using RADA16 (as PuraStat; 3-D Matrix Europe SAS., France) as a topical GI hemostat was reported in a study of 100 subjects (mean age 69.3 years; 32% female) undergoing complex endoscopic resection (ER) (79 ESD, 21 EMR) procedures at a single United Kingdom medical center (Subramaniam et al., 2019). Surgeries comprised 48 esophageal, 31 colorectal, 11 gastric, and 10 duodenal sites, and 30% of the patients were on antithrombotic therapy. Mean lesion size was 3.7 ± 2.1 cm and the mean resection base area was 14.1 ± 16.5 cm^2^. RADA16 was used to control IPB that occurred in 64% of cases (33 esophageal, 5 gastric, 6 duodenal, 20 colorectal), and was applied prophylactically to cover the resection base in all 100 cases. Diathermy was employed when RADA16 alone did not completely stop bleeding. RADA16 was delivered through the endoscope channel via 3-mL pre-filled syringes connected to 1600-mm (gastric) or 2200-mm (colonic) custom catheters (PuraStat Nozzle System Type-E, Top Corporation, Tokyo, Japan). RADA16 alone was an effective hemostat in 75% of these cases (73% of venous type mild oozing-to-moderate bleeding, and 50% of spurting bleeds). Thus, while having some utility for adjunctively controlling spurting bleeds, SAP-based hemostasis is best suited for controlling mild-to-moderate surgical bleeding, and for prophylactically preventing delayed bleeding onset. Only a small volume of RADA16, mean 1.8 ± 1.4 mL per lesion, was required for hemostasis of IPBs, whereas 2.6 ± 1.8 mL was used to completely cover the resection base. Hemostasis with RADA16 occurred in 70 ± 69 s. The delayed bleeding rate of 3% (two antral gastric cancer ESDs and one esophageal cancer ESD) was lower than anticipated in this high-risk cohort. Resection sites were clearly visible at all stages before, during, and after RADA16 application. The transparent and viscous nature of the RADA16 solution and resultant hydrogel permits its use as an adjunct therapy to traditional hemostatic methods such as clips or coagulation to control complex bleeding. There were no reported technical challenges such as catheter blockage and RADA16 was delivered successfully to the desired site in all cases with no technical challenges. No RADA16-associated AEs occurred.

The same United Kingdom group subsequently designed a randomized trial to compare RADA16 (PuraStat) adjunctive use (interventional group) against thermal coagulation-only (control group) for controlling IPB during ESD (Subramaniam et al., 2020). No other treatments (prophylactic coagulation or clipping) were performed in either group. This was a single-center study of 101 treatment-blinded subjects (*N* = 91 analyzed in the modified intention-to-treat cohort after exclusions and withdrawals) who underwent esophageal or colorectal ESD for lesions sized between 2 and 5 cm. Mean subject age was 71.5 ± 11.2 years in the diathermy-only control group (*n* = 45) and 68.6 ± 10.6 years in the RADA16 group (*n* = 46). Uninterrupted single antiplatelet therapy with aspirin was allowed, and all other anticoagulants were discontinued per local guidelines. Learning from their previous study (Subramaniam et al., 2019), grade 3 spurting bleeds were excluded and treated with other modalities. Fifty percent of the RADA16 group and 38% of Controls had significant comorbidities, and 40% of study participants had anticoagulant therapy stopped prior to their procedure. Lesion locations for the RADA16 group were 61% esophageal (*n* = 28) and 39% colorectal (*n* = 18), and 44% esophageal (*n* = 20) and 56% colorectal (*n* = 25) for the Control group.

The Control group had 269 IPBs in 45 subjects and RADA16 group experienced 232 IPBs in 46 patients (Subramaniam et al., 2020). The RADA16 group demonstrated a 50% reduction in the use of diathermy, with an overall 92.6% hemostatic effectiveness rate. Time-to-hemostasis was 70 ± 76 s in the RADA16 group and 78 ± 274 s in thermocoagulation-only Controls (*p* = 0.14). Total procedure times were similar in the RADA16 group at 74.2 ± 48.7 min and Controls at 80.7 ± 56.6 min. The RADA16 group received prophylactic hydrogel application to the resection base, and there was no difference in delayed bleed rates from lesions between the two groups (≈4% in both groups). Resection base healing was a secondary study end point and was significantly faster in RADA16-treated subjects than control subjects (detailed below in section “RADA16 to Enhance Wound Healing and Tissue Regeneration”).

Acute gastrointestinal bleeding (AGIB) is associated with significant morbidity and mortality (Gralnek et al., 2015). Depending on the cause and location of bleeding, managing AGIB can prove challenging. A recent retrospective observational study performed at three Italian hospitals evaluated endoscopically applied RADA16 (as PuraStat; 3-D Matrix Europe SAS) as an adjunct hemostatic method in 77 patients (41 upper GI, 36 lower GI) with AGIB (de Nucci et al., 2020). Cases in which RADA16 was used for primary hemostasis or prophylactically to prevent delayed bleeding after polypectomy, EMR, or ESD were excluded. The primary outcome was immediate hemostatic effectiveness with a secondary outcome being re-bleed rate within 7 days. Bleed types were defined as either spurting (*n* = 13) or oozing (*n* = 64). Sixty-five percent (50/77) of patients had an iatrogenic bleed, primarily as delayed bleeds following EMR (*n* = 29) and IPBs during EMR (*n* = 14). Of the 27 cases with non-iatrogenic bleeds, most were due to peptic ulcer (*n* = 19) and cancer (*n* = 5). Immediate hemostasis was achieved in 90% (70/77) of cases using adjunctive RADA16. Hemostatic failure was observed in 7 of the 13 spurting bleeds, comprising duodenal ulcer (*n* = 2), gastric ulcer (*n* = 2), and post-EMR (*n* = 1 each for colonic, gastric, and duodenal sites). Of these seven spurting-bleeding cases, bleeding was slowed enough with RADA16 in five cases to allow hemostasis achievement by another approach. The rebleeding rate within 7 days post-operatively was 10% (8/77 patients), consisting of gastric cancer (*n* = 3), gastric ulcer (*n* = 2), post-EMR gastric bleeds (*n* = 2), and duodenal ulcer (*n* = 1). Six of the eight rebleed patients were stabilized after RADA16 re-application, and the remaining two patients (both cancer) underwent surgery for bleeding. These data suggest the likely utility of RADA16 for treating AGIB, which will be assessed in more depth in planned prospective trials.

### RADA16 Hemostasis in Gastrointestinal Laparoscopic Surgery

Post-operative lymphorrhea can occur following lymphadenectomy during pelvic laparoscopic or open surgery (Ghezzi et al., 2012). Colorectal dissection often exposes large raw pre-sacral surfaces and can transect lymphatics and blood vessels, causing fluid leakage into the peritoneal cavity (White et al., 1985). Excess peritoneal fluid is usually reabsorbed; however, it can cause AEs including secondary infection from colorectal anastomosis leakage, sepsis, and thrombosis from vascular compression (Rudralingam et al., 2017). Several studies have investigated the utility of hemostatic RADA16 during laparoscopic colorectal resection, to staunch bleeding and exudative fluid flow into the peritoneal cavity.

A single-center Japanese study of 20 subjects that underwent laparoscopic pelvic surgery for rectal cancer reported reduced peritoneal effusion in the half of subjects (*n* = 10) who received 1% RADA16 (as PuraMatrix) treatment (Kondo et al., 2014). RADA16 was applied to the bleeding wound at 2 mL/5-cm^2^ surface area, twice, and this procedure was repeated if hemostasis was not initially achieved. Both groups were statistically similar in age, disease stage, and tumor location. Hemostasis was achieved within 5 min in all 10 RADA16-treated individuals. Operation time, bleeding volumes during surgery, hospitalization duration, and time with drain insertion were similar in both groups. Subjects receiving 1% RADA16 displayed significantly less post-operative pelvic drainage fluid (≈170 mL) compared with control subjects (≈350 mL; *p* < 0.01). No AEs occurred during the 2–3-month follow-up that were related to RADA16. The researchers concluded that 1% RADA16 was a safe and effective sealing material for preventing lymphorrhea after pelvic surgery.

A prospective observational study of 20 consecutive subjects (mean age 61 ± 2 years, range 52–70 years; 40% female) who underwent laparoscopic colorectal surgery was performed at one United Kingdom medical center (Ortenzi and Haji, 2020). Procedures included anterior resection (55%), sigmoidectomy (25%), and right hemicolectomy (20%). RADA16 (as PuraStat) was used as a method of hemostasis when conventional methods such as pressure application or thermal ablation were either insufficient or not recommended due to proximity to the ureter, pelvic/sacral veins, or other delicate structures. Mean overall surgery time was 185 ± 45 min. Mean RADA16 application time was 40 ± 17 s, and mean time to achieving hemostasis was 17.5 ± 3.5 s after treatment. Wound beds averaged 5.4 ± 2.3 cm^2^. No RADA16-related post-operative complications were observed; no delayed post-operative bleeding occurred.

In an abstract presented at the 2019 European Colorectal Congress, robust hemostasis was reported when RADA16 (PuraStat) was used in laparoscopic and robotic bowel resections (Stefan et al., 2019). Of 20 consecutive rectal cancer surgical cases, 10 received RADA16 adjunctive intraoperative hemostasis; controls used traditional hemostatic approaches only. Median drain output on Day 1 was 70 mL in the RADA16 group compared with 103 mL in the No-RADA16 group, indicating superior control of wound oozing and fluid exudate accumulation with the hydrogel. Median hospitalization duration was 5 days in both groups. Post-operative septic complications occurred in one RADA16 case and four conventional hemostasis-only cases. Post-operative ileus occurred in two RADA16 subjects and five patients in the No-RADA16 group. R0 resection was achieved in all patients. The authors appreciated the utility and effectiveness of RADA16 in significantly reducing pelvic oozing and bleeding after rectal cancer surgery.

### RADA16 Hemostasis for Radiation-Induced Proctitis

Radiation therapy is a common treatment modality for diverse pelvic cancers including prostate, bladder, cervical, uterine, rectal, and anal malignancies (Trzcinski et al., 2018). Radiation proctitis or proctopathy (RP) is a radiation-induced injury most commonly to the rectum seen in patients who have undergone pelvic radiotherapy (Tabaja and Sidani, 2018). Radiation proctitis can present as an acute inflammatory response involving only the superficial mucosa, or as a chronic pathogenic change in bowel function due to progressive endothelial dysfunction and vascular sclerosis, which includes bleeding, ischemia, and subsequent fibrosis (Weiner et al., 2016; White and Henson, 2021). Acute RP affects up to 75% of patients receiving pelvic radiotherapy, appearing within 1–6 weeks of treatment and with symptoms typically resolving within 3 months (Weiner et al., 2016). Chronic RP can occur months to years after radiation treatment, with a variably reported incidence ranging between 2.5 and 30%. Rectal bleeding is the most frequent symptom of chronic RP, occurring in 29–90% of patients (Tabaja and Sidani, 2018).

A report described a case series of 21 RP subjects (18 men; 17 prostate, 2 vaginal, 2 rectal; median age 76 years, range 47–84 years) who were administered RADA16 (as PuraStat) for hemostasis after rectal bleeding that remained refractory to standard treatment with sucralfate enema, hyperbaric oxygen therapy, and/or argon plasma photocoagulation (White and Henson, 2021). RADA16 was applied endoscopically at weekly intervals up to three times, with further treatments as determined by symptoms. Even in subjects with the most severe cases of RP, treatment with RADA16 improved self-recorded rectal bleeding amounts. Median episodes of bleeding reduced from 4.5 (range 0–27) to 2 (range 0–16) in the 7 days prior to the first and third treatment, respectively; Eight patients (38%) had no rectal bleeding following treatment, and 14 patients (67%) reported reduced bleeding episodes. Endoscopic grade defined by the Zinicola score (Zinicola et al., 2003) improved in 12 patients and 9 showed no change; however, 4/9 patients with no change in endoscopic grade started the study at the lowest grade and 5/9 with no grade change experienced a reduced number of bleeding episodes. Mean hemoglobin levels increased by 3.7 g/L from baseline to third treatment, and by 6.7 g/L from baseline to last follow up (median 12 months; range 3–18 months). Of patients followed up >12 months beyond their first RADA16 treatment, only one had recurrence of significant bleeding. Of 6 blood transfusion-dependent patients, four required no further transfusions; of the two patients that required additional transfusions, one had thrombocytopenia secondary to cirrhosis and the other had recurrent severe bleeding upon resuming aspirin monotherapy, which was then stopped.

Additional early data has been collected in a United Kingdom registry of prospective endoscopic studies to evaluate the role of RADA16 in diverse GI bleeding events, including RP. Initial data were presented at the 2019 European Society of Gastroenterology Congress detailed 226 procedures across three indications, including 22 RP cases (Arndtz et al., 2020). In these RP cases, RADA16 was used as a sole therapy in 14 individuals and secondary therapy in 8, and all resulted in a recorded improvement in patient-reported symptom score and measured hemoglobin levels. These results with RP cases support the expanded use of RADA16-based hemostasis to effectively treat bleeding sequelae arising from diverse iatrogenic causes, including pathologies arising from therapeutic radiation administration.

### RADA16 for Otorhinolaryngological Surgery

A case series report was the first to evaluate using RADA16 (as PuraStat) as a hemostatic agent in endonasal procedures (Lee et al., 2017). In that study, 60 subjects with severe allergic rhinitis and intractable nasal obstruction underwent endoscopic turbinoplasty by a single surgeon at three Australian hospitals. Remnant bleeding commonly occurs after turbinate resection surgery because the tissues are highly vascularized. This is typically an outpatient procedure and, after attempting conventional hemostatic approaches, patients are frequently discharged with nasal packaging tamponade to control bleeding. Nasal packing can cause discomfort, endonasal adhesions and infection, and delay healing (Fairbanks, 1986). One mL of RADA16 could cover an area of 1 cm^2^, so a single 5-mL syringe was sufficient for each bilateral turbinoplasty. The fluidity and transparent nature of the RADA16 solution allowed easy vertical application that ensured thorough coverage of the resected area while maintaining good wound visibility. No subject experienced adhesion formation or rebleeding, and all demonstrated normal operative site healing. Subsequently, another group reported the successful application of RADA16 for hemostasis in a 49-year-old man who underwent endoscopic endonasal surgery in Australia to divide a severe nasopharyngeal stenosis that arose secondary to chemoradiotherapy for squamous cell cancer of the tongue base (Wong et al., 2020). The patient was discharged the same day, having reported immediate post-operative improvement in subjective nasal patency. At 2 months follow-up, no rebleeding was reported by the patient, and complete resolution of his nasal obstructive symptoms was maintained. No evidence of recurrence or residual adhesion tissue was noted. These initial results suggest that RADA16 is suitable for consideration as an appropriate hemostatic agent in endonasal surgery. As mentioned previously, PuraSinus^TM^ has been cleared in the United States as a sinus hemostat but is also indicated for the prevention of adhesion formation and as an adjunct to wound healing (Figure 6).

## RADA16 for Wound Healing and Tissue Regeneration

Appropriate wound healing and tissue regeneration relies on a complex orchestration of multiple influences within the wound microenvironment, including cytokine, chemokine, and growth factor signals that recruit and activate cellular participants, vascularization and oxygenation status, mechanical forces, and the construction and remodeling of an ECM scaffolding (Rodrigues et al., 2019). The composition and structure of synthetic mesh-like ECM biomimetics, such as the interwoven fibers of the RADA16 hydrogel, may provide a favorable template for the repair of tissues damaged by surgery, pathology or trauma, including those of skin, bone, nerve, heart, liver, and other organs (Figure 6; Yi et al., 2017; Lee et al., 2019; Wang et al., 2019; Gelain et al., 2020).

Support for using RADA16 as an ECM surrogate for wound healing has been strengthened by numerous *in vitro* models in which RADA16 hydrogel supported proliferation, and differentiation of diverse cell types (Kakiuchi et al., 2013; Wang et al., 2019; Song et al., 2020). The simple peptide structure of RADA16 allows easy addition of various chemical moieties, which can then be rapidly screened for functional effects in wound-healing models (Bradshaw et al., 2014). An elegant 3D laboratory model of human skin was created by embedding human dermal fibroblasts and keratinocytes within a collagen type-I matrix (Schneider et al., 2008). Punchouts of these model tissue constructs underwent re-epithelialization at an accelerated rate when overlaid with 1% RADA16 hydrogel compared to healing observed without treatment, and even more so when epidermal growth factor was embedded within the RADA16 matrix. Thus, in addition to assisting wound healing by providing a physical 3D scaffold resembling native ECM, the RADA16 nanoporous matrix might also serve as a useful reservoir for the regulated release of therapeutic drugs and biologics (Figure 4).

Cartilage and bone defects might also be amenable to RADA16-mediated regenerative approaches. For example, chondrocytes embedded in RADA16 hydrogels maintained their differentiated status and expressed collagen type-II and glycosaminoglycans, with expression continuing to intensify through 3 weeks of culture (Liu et al., 2010). Scaffolds of RADA16 hydrogel containing co-embedded osteogenic adipose-derived stem cells and endothelial adipose-derived stem cells in a 1:1 ratio demonstrated both strong osteogenic and angiogenic differentiation, as gauged by expression of multiple cell-specific markers (Yang et al., 2018). Thus, RADA16 may serve as a useful scaffold for treating damaged or eroded cartilage and bone defects.

Animal studies have also supported a promising role for RADA16 not only as a hemostatic agent, but to facilitate wound healing, tissue regeneration, and angiogenesis (Cheng et al., 2013a, b; Wang et al., 2017, 2019; Gelain et al., 2020; Han et al., 2020). In a rat model of middle ear mucosal damage, cultured middle-ear mucosal epithelial cells were able to successfully repopulate and heal mucosal defects when cells were administered within a 2.5% RADA16 hydrogel matrix (as PuraStat), but instillation of cells suspended in culture media alone were unable to survive and repair the damaged mucosa (Akiyama et al., 2013). Purposefully created periodontal defects in rats showed significant healing and bone regeneration at 4 weeks when defects were initially filled with 2.5% RADA16 solution compared to defects filled with Matrigel (a mouse-derived ECM-like product) or left unfilled (Takeuchi et al., 2016). In a rat model of inflammatory colitis simulated by chemically induced colon mucosal ulceration, topical application of 1% RADA16 (as PuraMatrix) significantly suppressed colonic injury as viewed by endoscopy, downregulated expression of inflammatory cytokines, and increased expression of wound-healing factors (Araki et al., 2021). Topical application of RADA16 in an assessor-blinded porcine EMR study suggested enhanced neomucosal coverage and less submucosal damage with RADA16 versus no-RADA16 treatment, at 6 days after lesion creation (Tsiamoulos et al., 2017). Furthermore, a more recent study demonstrated that RADA16 reduces the incidence of esophageal stricture after 5-cm circumferential ESD in a porcine model by facilitating re-epithelialization (Oumrani et al., 2019, 2021). In a rat bone defect model, RADA16 formulations supported osteoregeneration, and showed utility as a depot for fibroblast growth factor (He et al., 2017). Human-induced pluripotent stem cells were encapsulated within RADA16 microspheres before induction toward a neural phenotype and transplantation into the brains of immunocompromised mice (Francis et al., 2016). The RADA16-encapsulated neurons displayed robust survival and neurite outgrowth *in vivo*, outnumbering surviving neurons that had been transplanted in suspension by two orders of magnitude. When injected at the injury site in rats with transected sciatic nerves, RADA16 hydrogel supported axonal outgrowth from damaged neurons, and the addition of functional motifs to the peptide monomers enhanced this activity. Similarly, the RADA16 meshwork is a permissive milieu for axonal regrowth from the severed optic tract of adult hamsters, sufficient to restore vision at least partially (Ellis-Behnke et al., 2006b). These observations suggest that RADA16 and related SAPs might deserve consideration as potentially effective reprogramming and transplantation vehicles for neurons and other cell types in future regenerative medicine studies (Figure 6).

Peptide chemistry allows the precise attachment of various functional moieties to RADA16 to confer novel biological activities to the hydrogel including enhanced tissue regenerative capacity. For example, GRGDS and YIGSR are peptide sequences unique to the ECM proteins fibronectin and laminin-1, respectively, which mediate cell adhesion to the matrix. In a rat liver injury model, application of 1% RADA16, either alone or coupled to either ECM moiety, resulted in more extensive wound healing after 2 weeks versus thermocautery control sites (Cheng et al., 2013a). The number of proliferating hepatocytes was greater in wounds managed with RADA16 coupled to either ECM sequence than in the RADA16-alone or control groups. In this model, dissolution of all three hydrogel scaffolds was obvious within 3–7 days and was complete by 2 weeks. In a rat traumatic brain injury model (forebrain punch biopsy), neural stem cells were suspended in either plain 1% RADA16 or in RADA16 coupled to another functional laminin motif IKVAV, and suspensions were applied to wound sites to polymerize (Cheng et al., 2013b). Addition of the ECM motif to RADA16 enhanced stem cell differentiation in hydrogels toward a neuronal phenotype and minimized astrocyte formation at 3 and 6 weeks compared to cells encapsulated in unmodified RADA16, suggesting enhanced regenerative capacity in functionalized RADA16 hydrogels. Histological analysis of the wound sites revealed hydrogel presence at 1 week, which was not visible at 3 weeks suggesting complete scaffold dissolution by this time. A subsequent study demonstrated enhanced endogenous neural stem cell differentiation, neurogenesis and functional recovery, and neovascularization in a zebrafish brain injury model when 1% RADA16 alone or coupled to SVVYGLR, an angiogenic α-integrin-binding sequence derived from osteopontin, was applied to wound sites (Wang et al., 2017). Thus, diverse pre-clinical experiments have demonstrated the unique potential of RADA16 and several of its variants in facilitating the recruitment, proliferation, and differentiation of multiple cell types, resulting in effective wound healing, neurogenesis, and angiogenesis. Future trials will capitalize on these observations to explore potential new clinical uses for RADA16 as a surrogate ECM in human regenerative medicine.

Several of the clinical trials mentioned earlier also explored RADA16 effects beyond achieving hemostasis, including observations on wound healing in humans. Gastric ESD ulcer healing by was assessed by evaluating mucosal coverage and margins of the ulcer (Uraoka et al., 2016), according to the Sakita classification (Sakita et al., 1971). An increased healing rate was observed after 1 week, and scarring was more extensive than anticipated between 4 and 8 weeks post-operatively, leading the authors to conclude that RADA16 might improve healing of ESD-induced gastric ulcers. In the randomized control trial that explored RADA16 use during esophageal and colorectal ESD (Subramaniam et al., 2020), resection base healing was a secondary study end point. At 4 weeks post-procedure, 75% of RADA16 ulcers showed either complete wound healing or scarring compared with only 54% of ulcers in the diathermy-only group. At Week 4, significantly more RADA16 subjects, 49%, achieved complete wound healing compared with 25% of control subjects. In a series of individuals who underwent bilateral turbinectomy, RADA16 was associated not only with effective hemostasis but also with the lack of adhesion formation during healing (Lee et al., 2017). Taken together, it appears that the porous meshwork of the RADA16 hydrogel not only functions as an effective barrier to bleeding, but also might support wound healing by acting as a temporary scaffold that facilitates the integration, proliferation, and maturation of the cells needed to create new tissue. Incorporating specific structural modifications into the RADA16 molecule might allow us to tailor hydrogel biological activities to provide optimal regenerative environments for different clinical applications.

## RADA16 Hydrogels as Drug Reservoir and Delivery Systems

The RADA16 nanoporous matrix might also serve as a useful reservoir for the regulated release of therapeutic drugs and biologics, to enhance various biological processes such as angiogenesis and neurogenesis that are important in wound healing and tissue regeneration. Sections of surrogate human skin tissue were created *in vitro* by embedding human dermal fibroblasts within a collagen type-I matrix and overlaying these with a stratified layer of keratinocytes (Schneider et al., 2008). After punch-out “wounding” of the skin construct, a drop of 1% RADA16 suspension was placed into and above the wound to polymerize. Inclusion of recombinant epidermal growth factor (10 μg/mL) in the RADA16 hydrogel caused an ≈4-fold increase in re-epithelialization over the wounded region versus RADA16 alone. Sequential measurements of the tissue culture media indicated that growth factor was released from the RADA16 hydrogel in a linear fashion through 24 h, at which point 65% of incorporated cytokine had been released, and significant addition growth factor egress from the hydrogel was not observed afterward. Furthermore, in a rat myocardial infarction model, injection of 1% RADA16-II (RARADADARARADADA) suspension containing platelet-derived growth factor-BB (PDGF) into the infarct border zone immediately after infarction was cardioprotective and resulted in improved cardiac function and reduced cardiomyocyte apoptosis measured 2 weeks post-infarction (Hsieh et al., 2006). Myocardial cell proliferation, neovascularization, regional blood flow, and local inflammation were not changed by RADA/PDGF treatment. Without the SAP scaffold, PDGF-BB injected alone rapidly disappeared from injected sites within 24 h, and only a negligible amount of PDGF-BB could be detected after 3 days. In contrast, when PDGF-BB was co-administered with RADA16-II, the resultant hydrogel scaffolding facilitated controlled growth factor release, and 16.1 ± 2.4% of PDGF-BB remained at the targeted delivery sites after 14 days.

Converse to regenerative and wound healing applications, RADA16 has also been explored as a potential delivery vehicle for antineoplastic agents, to destroy solid tumors. Syringe-delivery of a liquid therapeutic/RADA16 mixture directly into a solid tumor might polymerize into a biodegradable hydrogel that restricts gradual drug release to the local environment. This might be a promising approach to effectively obliterate individual tumors while minimizing the systemic toxicity associated with many cancer drugs. When the potent antitumor drug paclitaxel was embedded in RADA16 matrices, drug release kinetics were dependent upon RADA16 concentrations used to form the hydrogels, and controlled release from 1% RADA16 markedly inhibited proliferation of the human breast cancer cell line MDA-MB-435S for at least 1 week (Liu et al., 2011). Another group performed pre-clinical studies to explore the idea of loading RADA16 with tamoxifen and injecting it directly within the breast following lumpectomy, to exert cytotoxicity on any remnant breast cancer cells (Wu et al., 2017). Other peptide-based hydrogels and nanogels have also been explored *in vitro* to deliver doxorubicin, an antineoplastic drug used in stomach, bladder, breast, lung, and ovarian cancer therapies (Gallo et al., 2021).

Structural modifications to RADA16 can alter its function as a hydrogel drug delivery system. One group created hydrogels out of two differently modified RADA16 peptides with different functions (Huang et al., 2019). One peptide was end-linked to a QLK motif that facilitates crosslinking of the peptides by transglutaminase activity. Microbial transglutaminase was used to mimic the bioactivity expected from the intrinsic coagulation factor XIIIa, an endogenous enzyme activated by thrombin cleavage. Subsequent covalent crosslinks are resistant to proteolytic degradation thus providing increased mechanical stability of the hydrogel. The second RADA peptide was modified by the attachment of an LRK moiety, which regulates binding to heparan sulfates that are integral glycosaminoglycan components of native ECM. Importantly, heparan sulfates form non-covalent complexes with potent angiogenic molecules such as vascular endothelial growth factor (VEGF) and growth promoters such as hepatocyte growth factor (Rajangam et al., 2006). *In vitro*, enzyme crosslinking of 2% RADA16-QLK/LRK resulted in a 5-fold increase in hydrogel rheological stiffness. Crosslinked hydrogels were more resistant to degradation, showing 20% dissolution in 3 days versus 40% in non-crosslinked gels. At the 35-day study terminus, 61% of crosslinked and 82% of control gel volumes were degraded. Modified RADA16 hydrogels spiked with heparan-binding cytokines before polymerization displayed reproducible release kinetics *in vitro*. With VEGF, 32% of total cytokine was released within 3 days, and 62% through 28 days. In a chick chorioallantoic membrane assay, the modified RADA16 formulation resulted in significantly greater angiogenesis with larger-caliber vessels, when the applied hydrogel contained angiogenic growth factors. Thus, two minor RADA16 modifications resulted in significantly strengthened hydrogels with sustained growth factor release over 4 weeks. In a rodent myocardial infarction model, RADA16 was modified by attachment of a LRK-containing sequence and injected with VEGF into infarction-damaged hearts resulted in increased angiogenesis, better cell survival, less scar formation, and improved cardiac function at 1 month than injecting unmodified RADA16 plus VEGF (Guo et al., 2012). Such studies demonstrate the feasibility of modulating RADA16 structure to sequester and regulate the controlled release of therapeutic agents that drive wound healing and tissue regeneration.

Release kinetics of cytokines and other therapeutic agents from RADA-based hydrogels can likely be fine-tuned by altering the peptide concentration and chemistry (Gelain et al., 2020). Thus, in addition to assisting wound healing by providing a physical 3D scaffold resembling native ECM, both unmodified and structurally tailored RADA16 hydrogels might have unique abilities to direct specific tissue healing and reconstruction events through the selective presentation and release of incorporated biologics (Lee et al., 2019; Wang et al., 2019; Gelain et al., 2020).

## Potential Limitations of RADA16 Hydrogels

For hemostatic applications, RADA16 formulations are currently approved as topical agents to halt oozing and low-pressure bleeding. Unmodified RADA16’s shear-thinning/thixotropic nature probably precludes its utility in high shear environments (e.g., intra-arterial) where strong hemodynamic and rheological forces might inordinately increase the hydrogel’s disassembly rate. Hydrogel resistance to shear stress can be increased by either increasing the peptide concentration to form more durable hydrogels or by modifying the RADA16 structure by attaching motifs that enhance crosslinking or adhesion to surrounding tissues or endogenous coagulation components to provide additional anchoring strength. Still, for currently approved hemostatic applications, predictable RADA16 hydrogel dissolution to non-toxic metabolites occurs comfortably after the risk of delayed bleeding has passed and is a desirable trait.

As a surrogate ECM scaffolding for facilitating wound healing and tissue regeneration, the ability of RADA16 hydrogels to fill tissue voids and easy syringe and catheter application to difficult-to-reach sites are positive attributes. In some highly mobile or load-bearing sites such as joints and bones, different resident tissues and structures (e.g., tendon/sheath appositions within tendon sheaths, ligament insertions, long bone appositions after repair/reconstruction) are subject to variable and often strong mechanical forces. In these environments, the limited mechanical strength of RADA16-based hydrogels might risk damage to the structural integrity of the surrogate hydrogel ECM, and potentially compromise or delay regenerative outcomes. Although modifications to the RADA16 peptide concentration or chemistry might appreciably increase intrinsic gel strength and strengthen hydrogel attachments to surrounding tissues, hydrogel-based wound healing/regenerative approaches will still probably remain suitable only for environments where physical forces are either not extreme or can be temporarily restrained, for example by splinting/casting/anchoring (joint and long bone applications) or suturing (soft but mobile tissue applications).

Alternatively, to strengthen RADA16-based hydrogels, RADA16 might be co-polymerized with more mechanically durable polymeric scaffold materials. For example, composite nanoscaffolds created with functionalized RADA16 blended and co-polymerized within electrospun poly (L-lactic-co-glycolic acid) nanofibers generates a 3D matrix that displays stronger mechanical properties and slower degradation than RADA16 alone and promoted expression of molecular indicators of nerve repair and cell survival in an *in vitro* rat Schwann cell model (Nune et al., 2016). Similarly, a composite of 1% RADA16 modified with an RGD integrin binding motif was polymerized interspersed with a 5% photocrosslinkable polyester nanofiber suspension, and the resulting hydrogel was nearly three-fold stronger (storage modulus, G′) than 1% RADA16-only hydrogels and also had a reduced degradation rate (Zhai et al., 2020). Additionally, this combination improved wound healing in a rat spinal cord transection model. The ability to easily modulate RADA16-based hydrogel structure, cell and molecular interactions, and physical durability offer diverse possibilities for creative design of new hemostatic, tissue regenerative, and drug delivery platforms.

## Conclusion

Synthetic SAPs, exemplified in this review by RADA16, are unique in both character and in potential clinical utility. The ability of SAPs to spontaneously form higher-order structures, and our growing aptitude at customizing these peptides to selectively modify their polymeric architecture and associated biological interactions, together allow diverse new opportunities for biomedical exploration and therapeutic development. RADA16 has proven utility as an effective topical hemostatic agent in a variety of surgical and pathological scenarios. Now, accelerating research continues to clarify the functionality of RADA16 as a surrogate ECM that may have meaningful practical implications for wound healing in diverse organs. Additionally, the permissive microenvironment provided by the RADA16 nanofiber meshwork might be harnessed and modified to optimally synchronize the complex mechanics of cell and tissue development that are necessary for regenerative medicine applications. The unique and customizable nature of RADA16 and other SAPs also provides new prospects for use as a depot and targeted delivery system for drugs and biopharmaceuticals.

## Author Contributions

All authors were involved in all facets of literature review, manuscript construction and content, and critical revisions and approved the submitted version.

## Conflict of Interest

SS, KO’N, FR, MB, EA, MF, NM, EG, and LS are employees of 3-D Matrix, Ltd. MBD’A is a consultant for 3-D Matrix, Ltd.

## Abbreviations

3D: 3-dimensional
AE: adverse event
AGIB: acute gastrointestinal bleeding
ECM: extracellular matrix
EMR: endoscopic mucosal resection
ESD: endoscopic submucosal dissection
GI: gastrointestinal
IPB: intraprocedural bleeding
RADA16: (arginine-alanine-arginine-aspartic acid)_4_
RP: radiation proctopathy
SAP: self-assembling peptide
VEGF: vascular endothelial growth factor.
